# “Sightblind”: Perceptual Deficits in the “Intact” Visual Field

**DOI:** 10.3389/fneur.2013.00080

**Published:** 2013-06-25

**Authors:** Michał Bola, Carolin Gall, Bernhard A. Sabel

**Affiliations:** ^1^Medical Faculty, Institute of Medical Psychology, Otto von Guericke University of MagdeburgMagdeburg, Germany

**Keywords:** hemianopia, visual fields, perimetry, blindsight, vision disorders

## Abstract

Unilateral visual cortex lesions caused by stroke or trauma lead to blindness in contralateral visual field – a condition called homonymous hemianopia. Although the visual field area processed by the uninjured hemisphere is thought to be “intact,” it also exhibits marked perceptual deficits in contrast sensitivity, processing speed, and contour integration. Such patients are “sightblind” – their blindness reaches far beyond the primary scotoma. Studies showing perceptual deficits in patients’ intact fields are reviewed and implications of these findings are discussed. It is concluded that consequences of partial blindness are greater than previously thought, since perceptual deficits in the “intact” field likely contribute to subjective vision loss in patients with visual field defect. This has important implications for vision diagnosis and rehabilitation.

## “Blindsight” and “Sightblindness” – Peculiarities of Perception in Patients with Visual System Damage

Lesions of the visual system result in blindness in parts of the visual field retinotopically corresponding to the damaged tissue. Such visual field loss significantly impairs patients’ vision-related quality of life (Gall et al., 2009). Patients with unilateral visual cortex damage caused by trauma or posterior artery stroke are typically blind in the contralesional half of the visual field. However, the ipsilesional visual field, processed by the intact hemisphere, is considered intact and fully functional. Therefore, contralesional blindness is thought to be the main, if not only, cause of subjective visual impairment.

**Figure 1:**
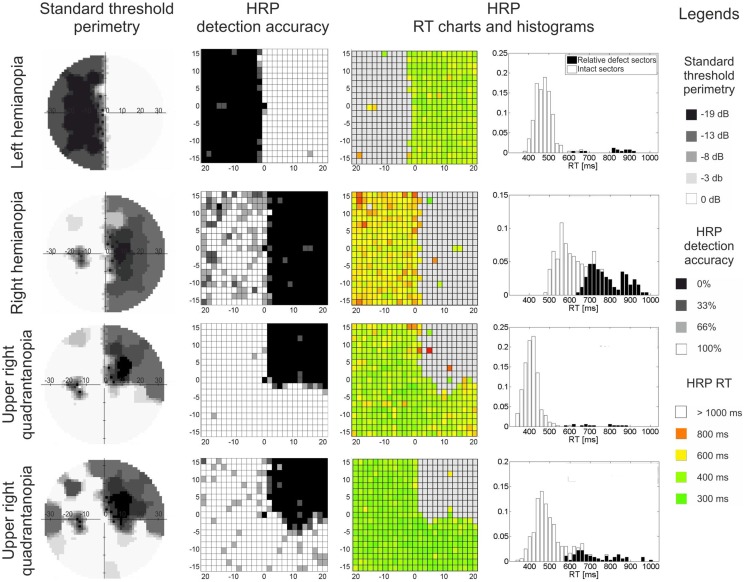
**Visual field maps of four patients with visual field loss due to post-chiasmatic damage**. Two types of visual field maps are shown: standard perimetry and high resolution perimetry (HRP), both showing comparable topographies of the visual field. In both methods, the visual field can be divided into absolute defect areas (black sectors, no detection), relative defect areas (also termed area of residual vision; gray sectors, partial detection, or elevated thresholds), and intact field (white sectors, full detection, low threshold). RT charts and RT histograms show processing speed deficits: (i) patients vary with respect to processing speed in the intact field, and (ii) residual vision areas are impaired when compared with intact sectors. Eccentricity (in degrees of visual angle) is denoted on horizontal and vertical axes in threshold perimetry and HRP charts.

Visual field loss is typically measured by perimetry, where simple detection tasks are used to approximate the location and extent of the underlying anatomical damage (Roux et al., 2001). Here, based on contrast threshold values or detection rates, visual field sectors are classified as absolute defect, relative defect, or intact areas (Figure 1). The “absolute” defect (blind field, scotoma) is the area where the subject does not consciously detect any perimetric stimuli. In cortically lesioned patients such blind fields are usually found on the contralesional side. In contrast, in areas of “relative” defect some detection abilities for moving stimuli or stimuli with increased luminance remain. These are typically located at the border of the lesion, but they can also be found deep inside the blind field as “islands of vision” (Fendrich et al., 1992). Both are believed to be the functional representation of partially damaged tissue. Areas of relative defect have also been termed “areas of residual vision” because of their restoration potential (Sabel et al., 2011a). Finally, the visual field area where all perimetric stimuli are detected is considered “intact.”

Contrary to the assumption that the “intact” field is fully functional, there are indeed perceptual deficits in this part of the visual field. Despite being “normal” in detection ability, the visual field regions corresponding to the uninjured hemisphere are deficient when perceptual functions of patients are tested more thoroughly and compared to an uninjured control group. We now term this phenomenon “sightblindness,” leaning on the reverse situation of “blindsight,” where residual perceptual capacities exist deep in the field of “absolute” blindness (Pöppel et al., 1973; Weiskrantz et al., 1974; review Cowey, 2010). We now review the existing evidence of sightblindness and discuss possible mechanisms and implications.

## Perceptual Deficits in the “Intact” Visual Field

Several studies suggest that visual functions are impaired in the “intact,” ipsilesional visual field of subjects with unilateral cortical lesions (homonymous hemianopia). Firstly, in comparison to healthy controls, hemianopic patients exhibit elevated contrast thresholds in the ipsilesional visual field (Hess and Pointer, 1989). Secondly, in a task requiring detection of a luminance change on noisy background, performance of patients in their intact field was characterized by longer reaction times (RT) and more false positive responses when compared to normal controls (Rizzo and Robin, 1996). Thirdly, longer RT in a simple light detection task and a higher double pulse resolution threshold, i.e., the lengthening of the minimally perceivable temporal gap between two light pulses, are yet another signs of visual processing deficits in hemianopia (Poggel et al., 2011).

Sightblindness is also observed in tasks demanding more complex processing of visual information. Contour integration in the intact field was probed with a task requiring patients to detect a figure (square) composed of aligned Gabor patches embedded in a background of randomly distributed Gabor patches (Paramei and Sabel, 2008; Schadow et al., 2009). Compared to control subjects, patients needed longer presentation times to accurately detect the target stimuli and yet their detection accuracy was worse. Interestingly, in a study investigating detection and categorization of natural scene images in the spared central visual field of hemianopic subjects, patients with lesions of the right hemisphere were impaired in both tasks, while patients with left hemispheric lesions were impaired in the categorization task only (Cavézian et al., 2010; Perez et al., 2012). Further, hemianopic patients often report difficulties searching their environment with eye movements (Pambakian et al., 2000) leading to disorientation and problems in avoiding obstacles. Lack of visual input does not fully account for abnormal patterns of eye movements during visual search (Machner et al., 2009), and this might be yet another manifestation of perceptual or temporal processing deficits in the intact field, though this hypothesis needs further study.

Finally, a case study of a patient suffering from quadrantanopia indicates that the intact field adjacent to the scotoma might not represent the visual stimuli accurately (Dilks et al., 2007). The patient perceived presented shapes as elongated toward the scotoma, e.g. a square as a rectangle, and a circle as an ellipse, which was interpreted as a perceptual consequence of maladaptive visual cortex retinotopic remapping (review Wandell and Smirnakis, 2009).

## Discussion and Implications

The body of evidence for “sightblindness” only starts to emerge and further studies supporting these initial observations are necessary. However, if confirmed, the presence of the “intact” visual field deficits has significant implications for researchers and clinicians working with visually impaired subjects.

### Mechanisms of sightblindness

The neurophysiological alterations resulting from visual system damage that account for the “intact” field deficits still need clarification, yet there are several possible explanations. It has been already shown that synchronization evoked by a stimulus presented in the seeing field of hemianopia patients is compromised (Schadow et al., 2009) and that different neural networks are activated during visual tasks in hemianopia subjects than in healthy controls (Perez et al., 2012). We hypothesize that lesion-induced disturbance of interhemispheric interactions might be the key mechanism, as in the lesioned hemisphere visually induced activation is weaker (Goebel et al., 2001; Nelles et al., 2007) and delayed (Rossion et al., 2000; Schoenfeld et al., 2002) when compared to the uninjured hemisphere. Reduced and delayed activation in the lesioned hemispheres might hamper the interhemispheric functional connectivity and consequently synchronization in the uninjured hemisphere (Schadow et al., 2009).

Further, cortical lesions lead to retinotopic reorganization of the visual cortex (specifically: receptive fields plasticity) which takes place in the area adjacent to the scotoma (Gilbert and Wiesel, 1992; Eysel et al., 1999; Baker et al., 2005; Wandell and Smirnakis, 2009). Such receptive field reorganization is related to increased excitability, and indeed, in cortically lesioned patients the area near the scotoma exhibits hyperexcitability as probed with neurophysiological methods (Braun et al., 2001). Perceptual distortions presumably resulting from cortical reorganization were already presented (Dilks et al., 2007) and might explain deficits occurring in the vicinity of the lesion.

However, intact field deficits were observed to occur in the intact visual field area distant from the scotoma, e.g., processed by the uninjured hemisphere. It has been shown that the visual cortex lesion affects activity and connectivity of down-stream visual structures (Goebel et al., 2001; Schoenfeld et al., 2002; Nelles et al., 2007). Crucially, unilateral cortical lesions alter activity of visual cortical areas not only in the damaged, but also in the seemingly unaffected (uninjured) hemisphere, which has been shown in animal model (Rushmore and Payne, 2003) and in patients (Henriksson et al., 2007; Nelles et al., 2007). Changes in activity are related to modification of anatomical (Bridge et al., 2008) and functional connectivity (Silvanto et al., 2009) between both hemispheres. However, none of the studies related the physiological changes in the uninjured hemisphere to the perceptual functions in the ipsilesional visual field. Therefore, future studies must define how the reorganization of visual networks, including the uninjured hemisphere, affects perception, and whether (or when) it is adaptive or maladaptive.

The two mechanisms, retinotopic remapping in the scotoma vicinity and modifications of activity of down-stream visual structures, might affect the intact field in a local and global manner, respectively. Indeed, in patients with visual field loss the temporal processing speed in the intact field is related to its distance to the scotoma – the closer to the scotoma the stimulus is presented the longer the RT (Bola et al., 2013). We interpret this as a sign of a local, spatially constrained (retinotopic) influence of the scotoma. At the same time “intact” field performance is associated with the scotoma size – the larger the scotoma, the longer the RT in the intact field. This may be interpreted as a manifestation of a global, spatially non-specific, i.e., non-retinotopic, influence of the lesion.

The existence of such a “global” lesion effect raises the question whether perceptual deficits in patients are limited to the visual domain. In the reviewed studies perceptual deficits were manifested not only by slower RT, but also by worse detection accuracy of figures on a noisy background (Paramei and Sabel, 2008; Schadow et al., 2009), worse accuracy in detection/categorization task (Perez et al., 2012), and lower double pulse resolution (Poggel et al., 2011). Further, the retinotopic influence of the scotoma (see above) indicates that intact field RT deficits are at least to some extent specific to visual processing – otherwise the deficits should been evenly spread over the whole visual field. Therefore, our working hypothesis is that the perceptual deficits (e.g., RT slowing) are specifically visual or greater in the visual domain than in other domains, but this needs to be tested in greater details. However, it is conceivable that extensive brain lesion, although located in the brain areas typically considered visual, might cause general, non-specific slowing of information processing (manifested by longer RT), affecting other domains (auditory, motor) as well. At the same time, persistent visual field defect might lead to widespread changes in the brain, e.g., disturbance of synchronization, oscillations, or functional connectivity (e.g., Dai et al., 2012), causing non-specific slowing secondary to the loss of visual input. Further, because intact field deficits were found in both post-chiasmatic and pre-chiasmatic patients (Bola et al., 2013), this suggests that not only cortical lesions but also pre-chiasmatic lesions might cause intact field deficits as well, although we do not know if the mechanisms of this impairment is different. These hypotheses need to be tested in future studies.

In this respect, sightblindness has important implications for the planning of experiments. When studying the effects of visual system damage either in animals or visually impaired patients, in many experiments the unlesioned hemisphere serves as a reference point as it is presumed to be “normal.” In view of “sightblindness,” researchers should keep in mind that these reference points (control values) are also to some extent defective, possibly biasing the results. It may be that existing data and their interpretation may require reappraisal. To avoid such a bias in future studies with hemianopic patients performance in perceptual tasks should always be compared to uninjured controls.

### Clinical implications

The “sightblindness” concept shows that our understanding about patients’ vision loss can only be rather incomplete when basing it solely on perimetry results of the primary scotoma. By testing only very basic perceptual functions, namely detection of simple static dots on uniform background, perimetry underestimates the true extent of functional deficits, especially those related to everyday visual functions. Thus, tests of higher visual functions are to be included in standard vision examinations if valid and comprehensive diagnosis of vision loss is the goal (see also Raz et al., 2012).

Further, the intact field deficits are expected to influence subjective quality of vision. An “objective–subjective mismatch” (Sabel et al., 2011a) was observed in subjects with persistent visual field loss, as objective measures of blindness (scotoma size measured by perimetry) and subjective vision loss (measured by vision-related quality of life questionnaires) were only modestly correlated (Müller et al., 2003; Gall et al., 2009). This indicates the existence of factors other than scotoma size to account for the subjective visual impairment, and sightblindness is one possible candidate.

Therapeutic applications in vision rehabilitation do not typically aim at improving visual processing deficits in the intact field. Although activating residual structures is crucial for visual recovery and restoration (Sabel et al., 2011a), improving the quality of vision in the intact field sectors is expected to benefit patients as well. Therefore, measures of intact field functioning should also be included when testing vision restoration methods like behavioral trainings (Kasten et al., 1998; Poggel et al., 2004), non-invasive brain stimulation (Sabel et al., 2011b), or both combined (Plow et al., 2012).

Altogether, advancing our knowledge about perceptual deficits in the intact visual field may result in a better understanding of normal and abnormal visual system functioning; it is the “second face of blindness.” Measuring sightblindness is also an opportunity to improve diagnostic and therapeutic tools with the aim to maximize recovery of vision including these more subtle deficits. This will then better appreciate – and improve – the subjective suffering of patients with partial blindness caused by damage of brain structures and consider their vision impairment in a more holistic manner.

## Conflict of Interest Statement

The authors declare that the research was conducted in the absence of any commercial or financial relationships that could be construed as a potential conflict of interest.

## Abbreviations

HRP, high resolution perimetry; RT, reaction time.
